# The experiences of research participants offered genetic test results as a result of taking part in a population based ovarian cancer research study?

**DOI:** 10.1186/1897-4287-10-S2-A15

**Published:** 2012-04-12

**Authors:** MA Young, S Wake, K Alsop, D Bowtell, G Mitchell, L Plunkett, A Crook, M Gleeson, N Hallowell

**Affiliations:** 1 The Peter MacCallum Cancer Centre, East Melbourne, Victoria, Australia; 2 Department of Paediatrics, the University of Melbourne, Royal Children’s Hospital, Flemington Rd, Parkville, VIC, Australia; 3 Westmead Institute for Cancer Research, University of Sydney at Westmead Millennium Institute, and Departments of Gynaecological Oncology, Westmead Hospital, NSW, Australia; 4 Queensland Institute for Medical Research, Brisbane, QLD, Australia; 5 Hunter Genetics, Newcastle, NSW, Australia; 6 Newcastle University, Newcastle upon Tyne, UK

## Background

Although the issue remains controversial, it is generally accepted that researchers have some responsibility to notify participants of information discovered during research that has the potential to significantly affect person’s health or prevent significant harm. Whilst the issue of recontact has been broadly discussed by ethicists, researchers and clinicians, few studies have reported on participant’s experiences of the process.

The Australian Ovarian Cancer Study (AOCS), a population based study, recruited women with invasive ovarian cancer between 2002 and 2006. BRCA1 or BRCA2 mutation testing has been undertaken and women in whom a mutation has been identified, or their next of kin in the case where the women is deceased, have been notified in writing (notification letter) by the researchers about the finding of a mutation and the availability of obtaining these results through a family cancer clinic (FCC). The AOCS Psychosocial project has interviewed individuals who received notification letters.

## Aims of AOCS Psychosocial study

1. Explore individuals’ understanding and response to the information contained in the letter they received from the researchers.

2. Determine what informs individuals’ decisions about whether or not to contact an FCC and take up genetic testing information.

## Results

A total of 21 in depth interviews have been undertaken to date. Participant’s response to the notification letter and their understanding of the letter varied. Some participants did not recall receiving the letter. Although many of the participants made contact with an FCC after reading the letter, the data suggest some participants were confused or did not understand the notification letter. Some expressed fear or ambivalence about the contents of the letter. In addition, the invitation to participate in the psychosocial study and resultant interview process acted as an intervention, with three participants stating receiving the letter to participate in the psychosocial study made them reconsider contacting an FCC. Whilst the primary purpose of the psychosocial interview was for collecting research data, for three participants the interview enabled clarification of misinformation and subsequent referral to an FCC.

